# Placental Cadmium Levels Are Associated with Increased Preeclampsia Risk

**DOI:** 10.1371/journal.pone.0139341

**Published:** 2015-09-30

**Authors:** Jessica E. Laine, Paul Ray, Wanda Bodnar, Peter H. Cable, Kim Boggess, Steven Offenbacher, Rebecca C. Fry

**Affiliations:** 1 Department of Epidemiology, Gillings School of Global Public Health, University of North Carolina, Chapel Hill, North Carolina, United States of America; 2 Department of Environmental Sciences and Engineering, Gillings School of Global Public Health, University of North Carolina, Chapel Hill, North Carolina, United States of America; 3 Department of Obstetrics and Gynecology, School of Medicine, University of North Carolina, Chapel Hill, North Carolina, United States of America; 4 Department of Periodontology, School of Dentistry, University of North Carolina, Chapel Hill, North Carolina, United States of America; Stony Brook University, Graduate Program in Public Health, UNITED STATES

## Abstract

Environmental exposure to heavy metals is a potentially modifiable risk factor for preeclampsia (PE). Toxicologically, there are known interactions between the toxic metal cadmium (Cd) and essential metals such as selenium (Se) and zinc (Zn), as these metals can protect against the toxicity of Cd. As they relate to preeclampsia, the interaction between Cd and these essential metals is unknown. The aims of the present study were to measure placental levels of Cd, Se, and Zn in a cohort of 172 pregnant women from across the southeast US and to examine associations of metals levels with the odds of PE in a nested case-control design. Logistic regressions were performed to assess odds ratios (OR) for PE with exposure to Cd controlling for confounders, as well as interactive models with Se or Zn. The mean placental Cd level was 3.6 ng/g, ranging from 0.52 to 14.5 ng/g. There was an increased odds ratio for PE in relationship to placental levels of Cd (OR = 1.5; 95% CI: 1.1–2.2). The Cd-associated OR for PE increased when analyzed in relationship to lower placental Se levels (OR = 2.0; 95% CI: 1.1–3.5) and decreased with higher placental Se levels (OR = 0.98; 95% CI: 0.5–1.9). Similarly, under conditions of lower placental Zn, the Cd-associated OR for PE was elevated (OR = 1.8; 95% CI: 0.8–3.9), whereas with higher placental Zn it was reduced (OR = 1.3; 95% CI: 0.8–2.0). Data from this pilot study suggest that essential metals may play an important role in reducing the odds of Cd-associated preeclampsia and that replication in a larger cohort is warranted.

## Introduction

Cadmium (Cd) is a ubiquitous toxic metal currently ranked 7th on the priority List of Hazardous Substances by the Agency for Toxic Substances and Disease Registry [1]. Industrial activity such as non-ferrous metal mining and refining, manufacturing and application of phosphate fertilizers, fossil fuel combustion, and waste increases Cd leaching into the soil/water/air/food [2]. Cd can contaminate the soil/water/air/food by disposal of consumer products such as batteries, pigments, coatings, and plastics. Another source of Cd exposure is via cigarette smoke [2].

Chronic Cd exposure is associated with renal disease, hypertension, and cancer [3]. Cd exposure during pregnancy has the potential to harm both the mother and fetus, as the placenta does not serve as a complete barrier against Cd [4]. In support of this, maternal serum Cd levels are significantly associated with umbilical and neonatal Cd levels highlighting the transfer of this toxic metal to the fetus [5–7]. *In utero* exposure to Cd is associated with low birth weight, decreased neonatal length and head circumference, and impaired childhood neurobehavioral and physiological development [8]. In addition, mean placental levels of Cd have been shown to be higher in preeclamptics versus normotensive women [9]. However, the relationship between placental Cd levels and risk for preeclampsia is currently unknown.

There are known interactions between Cd and essential metals such as zinc (Zn) and selenium (Se). Specifically, Zn and Se are both anti-oxidants that protect against Cd-induced toxicity [10]. Also, increased placental Cd concentrations may impair essential elements such as Se and Zn transfer to the fetus [4]. This is of concern as Zn deficiency is associated with fetal growth restriction and neurodevelopment abnormalities [11, 12]. Furthermore, maternal Zn deficiency has been associated with preeclampsia [13]. Women with preeclampsia have been shown to have lower levels of Se compared to normotensive women [14–16]. The relationship between Cd, Se, and Zn levels in the placenta as they relate to of risk of preeclampsia is not known.

The aims of the present study were to measure placental Cd, Se, and Zn levels using samples from a pregnancy cohort representing women across the southeastern US and to examine associations of placental metal levels with the risk of preeclampsia. Biomonitoring of maternal exposure to metals will potentially help to reduce prenatal exposures to toxic metals and inform nutritional supplementation of essential metals, ultimately reducing future adverse pregnancy complications and birth outcomes.

## Materials and Methods

### Study Population

This study is a nested case-control study within the Maternal Oral Therapy to Reduce Obstetric Risk (MOTOR) study. This study was approved by the University of North Carolina, Chapel Hill Institutional Review Board. The MOTOR cohort was a randomized, treatment-masked, controlled clinical trial of 1,806 pregnant women with periodontal disease who were receiving standard obstetric care (ClinicalTrials.gov Identifier: NCT00097656) [17]. Written consent was obtained from all participants. The cohort has been fully described previously [17]. Briefly, from December 2003 through October 2007 subjects were enrolled at Duke University Medical Center (and the affiliated clinic at Lincoln Health Center), the University of Alabama at Birmingham Medical Center, and two obstetric sites of the University of Texas Health Science Center at San Antonio (University Health Center-Downtown of University Health Systems and Salinas Clinic of the San Antonio Metropolitan Health District). Randomization occurred between February 2004 and September 2007.

For this nested case-control study, 172 subjects were selected; 86 with preeclampsia and 86 healthy, normotensive women. Preeclampsia was defined using criteria current at the time of the original study [18] as newly diagnosed hypertension (blood pressure >140/90 mmHg on two occasions 6 hours apart) and proteinuria (>300 mg of protein in a 24 h urine collection or urine protein/creatinine ratio of 0.3 mg/dL) occurring at > 20 weeks’ gestation. Controls were randomly chosen among those without preeclampsia. Baseline maternal characteristics such as education, socioeconomic status (SES), and age, amongst others did not differ between the nested cohort and the parent cohort (data not shown).

### Placental Collection and Metals Measurements

Immediately after placental delivery, a single, full-thickness biopsy of the placenta was obtained and flash-frozen. Biopsies were taken from the central zone of the placenta and standardized across all samples as previously described [17]. Abnormal gross placental pathology (i.e. infarcted areas) was avoided. Harvested placental tissues were stored at -70°C.

Placenta samples (169.7 ± 33.1 mg) were digested with 70% nitric acid at room temperature for five hours before being placed in an 85 degree heating block overnight. After cooling to room temperature, 30% hydrogen peroxide was added and the samples were returned to the heating block. The vials were removed, cooled, and vented every twenty minutes for the first hour. Digestion continued for an additional four hours and was complete after 24 hours. The samples were then were diluted to 4 mL with deionized water. The determination of total Cd, Se, and Zn was performed using the Agilent Technologies 7500cx inductively coupled plasma mass spectrometer (ICP-MS), (Santa Clara, CA. USA). Analytes were quantified using published protocols [19]. Cd and Zn isotopes measured were 111 *m/z* and 66 *m/z* respectively. External calibration and quality control standards were prepared from National Institute Standards Technology (NIST) traceable solutions (High Purity Standards, Charleston, SC. USA). The detection limit reported was corrected for dilution and normalized to a typical sample mass of 0.150g. The limit of detection for the metals was: 1.5 ng/g (Cd), 1.5 ng/g (Se), and 300 ng/g (Zn).

### Statistical Analyses

The statistical package SAS 9.4 (SAS Institute Inc., Cary, North Carolina) was used to analyze the data. Spearman rank correlations were calculated to measure the inter-relationship among placental metals levels and were considered to be significantly correlated if *p<0*.*05*. Chi Square tests for differences in proportions of demographic variables between normotensives and preeclamptics were carried out; significance was set at *p<0*.*05*. Difference of means tests calculated for demographic variables were performed using a two-sided Student’s t-test with significance set at *p<0*.*05*. Wilcoxon rank sum tests were utilized to determine differences in the levels of Cd, Se, and Zn between normotensives and preeclamptics with significance set at *p<0*.*05*. To determine if Cd levels differed based on women’s self-reported smoking status for both pre pregnancy and during pregnancy smoking a Wilcoxon rank sum tests was performed. Unconditional logistic regressions were performed accessing odd ratios (OR) and 95% Confidence Intervals (CI) for PE with exposure to Cd (as a continuous variable) controlling for confounders, as well as interactive models of Cd x Se and Cd x Zn. Confounders were selected *a priori* based on their known associations with both the exposure (metals) and preeclampsia. Model 1 represents the unadjusted model without the inclusion of confounders. Model 2 included the following covariates: correlated metals (continuous variable), and demographic variables collected from in person questionnaires including, maternal age (continuous variable), education (trichotomized), race (dichotomized), tobacco and alcohol use during pregnancy (dichotomized as yes/no), public assistance recipient (dichotomized as yes/no as proxy of SES), gestational age (continuous variable), previous pregnancies (dichotomized as 0/>1), magnesium sulfate treatment (yes/no), and periodontal disease treatment (dichotomized as pre or post-delivery). For sensitivity analyses, two additional models were run as a variation of Model 2. Model 3 excluded gestational age as a covariate and Model 4 excluded periodontal disease treatment as a covariate. Interactive models of Cd x Se and Cd x Zn included an interaction term for Cd (continuous) x Se (dichotomized based on median levels of Se) and Cd (continuous) x Zn (dichotomized based on median levels of Zn). Interaction term significance was set at *p*<*0*.*05*. ORs were determined to be significant if the 95% CI did not include the null value of 1.

## Results

### Characteristics of the Nested Case-Control Study

Maternal and birth characteristics for the nested case-control study are presented in Table 1. The average age at delivery for all women was 25 years with 38% of the women self-identified as white and 62% as black. Most of the women had a high school education or greater (85%), received public assistance (73%), and reported no tobacco use during pregnancy (96%) (Table 1).

**Table 1:** Demographic Characteristics of the Nested Case-Control Study Participants.

Characteristic	Mean, Median [Range] or *n* (%)			
	All (n = 172)	Controls (Normotensives) (n = 86)	Cases (Preeclamptics) (n = 86)	Chi Square test statistic or T-Test (*p*-value)
**Maternal Age at Delivery (years)**	25, 24 [16–41]	25, 24 [16–41]	24, 23 [16–40]	(0.16)
**Race**				
White	66 (38)	33 (38)	33 (38)	
Black	106 (62)	53 (62)	53 (62)	0 (1)
**Education**				
< High School	25 (15)	13 (15)	12 (14)	
High School	109 (63)	53 (62)	56 (65)	
> High School	38 (22)	20 (23)	28 (21)	0.23 (0.89)
**Receiving government assistance**				
No	46 (24)	30 (35)	16 (19)	
Yes	126 (73)	56 (65)	70 (81)	5.8 (0.015)
**Smoking during pregnancy**				
Non-smoker	156 (96)	75 (95)	81 (98)	
Smoker	6 (4)	4 (5)	2 (2)	0.87 (0.34)
**Smoking pre-pregnancy**				
Non-smoker	145 (84)	73 (85)	72 (84)	
Smoker	27 (16)	13 (15)	14 (16)	0.044 (0.83)
**Alcohol Consumption**				
None	167 (97)	84 (98)	83 (97)	
Some	5 (3)	2 (2)	3 (3)	0.21 (0.64)
**Method of Delivery**				
Vaginal	115 (67)	58 (67)	57 (66)	
Caesarean section	57 (33)	28 (33)	29 (34)	0.026 (0.87)
**Gestational Age (weeks)**				
All	39, 39 [26–42]	40, 40 [37–42]	38, 38 [26–42]	(<0.001)
<37 weeks	23 (13)	0 (0)	23 (27)	
≥37 weeks	149 (87)	86 (100)	63 (73)	26.6 (<0.001)
**Newborn sex**				
Male	93 (54)	51 (60)	42 (49)	
Female	79 (46)	35 (40)	44 (51)	1.9 (0.16)
**Birth weight (g)**	3205, 3195[760–4940]	3420, 3335 [2515–4495]	2997, 2966[760–4940]	(<0.001)
**Low Birth Weight (LBW)**	17 (10)	0 (0)	17 (20)	
**Newborn Length (cm)**	50, 50 [33–57]	50, 51 [33–57]	49, 49 [33–55]	(0.0014)
**Head Circumference (cm)**	34, 34 [24–49]	34, 34 [31–49]	33, 34 [24–48]	(0.0130)
**Fetal Congenital Abnormalities**	7 (4)	5 (6)	2 (2)	1.3 (0.24)
**Previous Pregnancies**				
0	42 (24)	14 (16)	28 (33)	
≥1	130 (76)	72 (84)	58 (67)	6.2 (0.013)

### Placental Metals Levels

No metal levels were below the detection limit. The mean placental Cd level was 3.6 ng/g (median: 3.0; range: 0.52–14.5 ng/g; SD = 2.0). Mean placental Cd levels were higher among cases compared to controls (3.7 ng/g versus 3.5 ng/g, *p* = 0.44). The mean placental Se level was 246.4 ng/g (median: 248.4; range: 90.2–514.3 ng/g; SD = 89.7) with Se levels lower among cases compared to controls (237.5 ng/g versus 254.5 ng/g, *p* = 0.19). The mean placental Zn level was 8659.1 ng/g, (median: 8525.2; range: 3595.2–18573.9 ng/g; SD = 2105.6). Mean placental Zn levels were significantly higher in cases compared to controls (8892.0 ng/g versus 8414.7 ng/g, *p* = .04). Placental Cd and Se levels were both significantly correlated with placental Zn levels *(p*<0.001 and *p* < .05, respectively) (Table 2). In relationship to smoking status, placental Cd levels were slightly higher in smokers than in non-smokers but not statistically significant. The lack of significant difference may suggest other potential environmental sources for Cd (S1 Table).

**Table 2:** Levels and correlations of placental metals in all subjects, normotensives (controls), and preeclamptics (cases); all values are reported as ng/g wet weight.

	All (n = 172)		Controls (Normotensives) (n = 86)		Cases (Preeclamptics) (n = 86)	
	Mean (Median)	Range	Mean (Median)	Range	Mean (Median)	Range
**Cd (ng/g)**	3.6 (3.0)	[0.52–14.5]	3.5 (3.1)	[0.52–8.7]	3.7 (3.0)	[0.84–14.5]
**Se (ng/g)**	246.4 (248.4)	[90.2–514.3]	237.5 (208.2)	[91.5–514.3]	254.5 (269.7)	[90.2–445.6]
**Zn (ng/g)**	8659.1 (8525.2)	[3595.2–18573.9]	8414.7 (8175.4)	[3595.2–18573.9]	8892.0(8723.4)*	[4825.0–16368.8]
	Correlation Coefficient^+^ (*p*-value)		Correlation Coefficient^+^ (*p*-value)		Correlation Coefficient^+^ (*p*-value)	
**Cd—Zn**	0.36 (*p*<0.001)		0.38 (*p*<0.001)		0.35 (*p*<0.001)	
**Cd—Se**	-0.067 (0.10)		0.081 (0.45)		-0.20 (0.06)	
**Se—Zn**	0.19 (0.04)		0.30 (0.04)		0.079 (0.47)	

*p<0.05, Wilcoxon Rank Sum Test between cases and controls.

^+^ Spearman Rank Correlation Coefficient

### Odds Ratios of PE in Relationship to Cd, Zn and Se

The adjusted odds ratio (Model 2) for PE in relationship to placental Cd levels was 1.5 (95% CI: 1.1–2.2) (Table 3). The interaction terms of both Cd x Se, and Cd x Zn were significant (*p*<0.05). ORs for PE in relationship to Cd were assessed in relationship to lower or higher Zn or Se based on their median values of 8669 ng/g and 246 ng/g respectively. When an interaction term was included in the model for Cd x Zn, the OR for PE in relationship to placental Cd levels in placentas with lower Zn levels was 1.8 (95% CI: 0.8–3.9). In contrast, the OR for PE in placentas with higher Zn levels was 1.3 (95% CI: 0.8–2.0) (Table 3). When an interaction term was included in the model for Cd x Se, the OR for PE in relationship to elevated placental Cd level for placentas with lower Se levels was 2.0 (95% CI: 1.1–3.5). This differed from the OR for PE in relationship to higher Se calculated to be 0.98 (95% CI: 0.5–1.9) (Table 3). As gestational age may be a mediator of the relationship between Cd exposure and PE a sensitivity analysis (Model 3) excluding gestational age was carried out. The results demonstrated a reduction in the ORs of PE (S2 Table). As this study is a nested case-control within the MOTOR cohort with a focus on periodontal disease, a sensitivity analysis (Model 4) excluding periodontal disease treatment was assessed. The results demonstrated no change in the ORs (S2 Table). The minimal dataset used for analysis is provided (S1 Data).

**Table 3:** Odds ratios and 95% Confidence Intervals (CI) for preeclampsia in relationship to placental Cd, Se or Zn and interactive models for Cd with essential metals Se and Zn.

	Unadjusted Odds Ratio (95% CI)^a^	Odds Ratio (95% CI)^b^
**Cd**	1.1 (0.9–1.2)	1.5 (1.1–2.2)
**Se**	1.0 (0.99–1.0)	1.0 (0.99–1.0)
**Zn**	1.0 (1.0–1.0)	1.0 (1.0–1.0)
**Cd x Se**		
**Se ≤ median level** ^**+**^	1.1 (0.9–1.4)	2.0 (1.1–3.5)
**Se > median level** ^**+**^	0.98 (0.78–1.2)	0.98 (0.5–1.9)
**Cd x Zn**		
**Zn ≤ median level** ^**++**^	0.97 (0.71–1.3)	1.8 (0.8–3.9)
**Zn > median level** ^**++**^	1.0 (0.84–1.2)	1.3 (0.8–2.0)

^a^Model 1 represents the crude or unadjusted model.

^b^Model 2 correlated metals, maternal age, education, race, tobacco, alcohol use during pregnancy, public assistance recipient, gestational age, previous pregnancies, magnesium sulfate treatment, and periodontal disease treatment.

+Se median = 246 ng/g

++Zn median = 8669 ng/g

## Discussion

The placenta represents a unique temporary organ for the measurement of toxic and essential metals. It serves as the interface between fetal and maternal circulation facilitating the exchange of gases and transport of nutrients, but also as a potential source for fetal exposure to environmental contaminants such as Cd. It is currently unknown whether placental Cd levels are associated with risk for preeclampsia. In the present study we set out to establish whether Cd is associated with an increased risk in samples collected from women residing in the Southeastern US (Alabama, North Carolina, and Texas) using a nested case-control study design within the MOTOR cohort. The mean level of placental Cd level was 3.6 ng/g and ranged from 0.52 ng/g up to 14.5 ng/g. Supporting the broader relevance of the findings from this study, these placental levels are consistent with those observed both in the US as well as globally [20]. We found that placental Cd levels are significantly associated with preeclampsia (OR = 1.5; 95% CI: 1.1–2.2), highlighting the potential impact of toxic metal exposure on adverse pregnancy outcomes.

In addition to the measurement of Cd, we also examined placental levels of Se and Zn that were found to be comparable to other US cohorts [4, 19]. Interestingly, when analyzed in the context of placental Se and Zn levels the relationship between placental Cd exposure and preeclampsia was altered. Placental Se levels below the median significantly increased the association between placental Cd levels and preeclampsia resulting in an OR of 2.0 (95% CI: 1.1–3.5). This was in contrast to the observed OR when placental Se levels were above the median of 0.98 (95% CI: 0.5–1.9). These data suggest that placental Se protects against Cd-associated PE. Our findings contribute to a growing body of literature relating decreased maternal Se levels with PE [14–16] and that Se supplementation may reduce PE risk [21]. While the CI intervals included the null value for each level of Zn, the ORs indicate that lower placental Zn levels increased the odds for PE (OR = 1.89; 95% CI: 0.8–3.9) compared to the OR for Cd alone (OR = 1.5; 95% CI: 1.1–2.2). Our data compliment previous toxicologic studies that show Zn is protective against Cd-mediated toxicity and that Zn deficiency increases Cd toxicity [22]. It remains to be determined whether maternal serum or placental Zn insufficiency predisposes to placental Cd toxicity and development of preeclampsia or if Cd toxicity impairs Zn transfer and placental function leading to preeclampsia. This is the first study to demonstrate interactive effects of both placental Se and Zn in relationship to Cd-associated PE.

Possible biological mechanisms for the relationship between Cd-associated PE and subsequent protection from Cd toxicity by Se and Zn are suggested through *in vitro* studies and animal models. One mechanism for Cd-associated PE may be through oxidative stress and/or reactive oxygen species (ROS) as Cd induces both [23]. Oxidative stress, indicated by increased ROS in the placenta, is known to be associated with impaired placental function, pregnancy complications, and decreased size at birth [24–26]. Additionally, it has been suggested that the protective role of Zn against Cd-induced toxicity may be through the reduction of Cd-induced ROS [27]. The precise mechanisms by which Se and Zn protect against Cd-induced toxicity are not established.

While our findings suggest that placental Cd levels are associated with PE and that Se may be protective of such a relationship, there are potential limitations. These analyses are limited to Cd, Se, and Zn and it is known that there are other toxic and essential metal interactions that may influence Cd absorption and toxicity [28]. In relationship to smoking status, placental Cd levels were slightly higher in smokers than in non-smokers but not statistically significant. The lack of significant difference may suggest other potential environmental sources for Cd. Still smoking status information was collected via self-report and measurements of cotinine were not available. Additionally, while we have sufficient power to determine the risk of preeclampsia with placental Cd levels, the sample size may limit strong conclusions based on interactive models. Thus, to further support these findings replication in a larger cohort is needed. While the current study uses a cross-sectional measurement of placental Cd, it is a suitable exposure indicator as it is a cumulative toxicant with a multi-year half-life in numerous tissues [1]. Furthermore, measures of Cd in the placenta have been shown to correlate with levels in both maternal blood and urine [4, 29]. The relationships between placental Se and Zn and circulating levels of these essential metals are not established and should be analyzed in future work.

In summary, this is the first study to address interactive effects of the toxic metal Cd and essential metals in the placenta with odds of preeclampsia in a US cohort. Its strengths include the measurement of placental metals levels as an exposure biomarker for both maternal and fetal health. Such measures have been suggested as highly informative and practical application in birth cohorts [20]. An additional benefit of this study is an examination of the interactions between Cd, an environmental toxicant, and Se and Zn which are essential metals. Our data suggest that the interaction between these metals may be more biologically relevant than studying single metals alone. Overall the results from this study suggest that Se and Zn may play an important role in reducing the risk of Cd-associated preeclampsia. Future studies will benefit from addressing the role of essential metals in reducing the risks of diseases during pregnancy and the interplay with toxic metal exposure.

## Supporting Information

S1 Data Minimal dataset used for analysis. (XLSX)

S1 Table Placental Cd levels (ng/g) of controls (normotensives) and cases (preeclamptics) based on self-reported smoking status both prior to or during pregnancy. (DOCX)

S2 Table Odds ratios and 95% Confidence Intervals (CI) for preeclampsia in relationship to placental Cd and interactive models for essential metals Se or Zn. (DOCX)
