# Supplementary material for: Placental Cadmium Levels Are Associated with Increased Preeclampsia Risk
Source: PLoS One. 2015 Sep 30;10(9):e0139341. doi: 10.1371/journal.pone.0139341 (PMC4589375; doi:10.1371/journal.pone.0139341)
Supplement: S1 Table — (DOCX) [file pone.0139341.s002.docx]

S1 Table. Placental Cd levels (ng/g) of controls (normotensives) and cases (preeclamptics) based on self-reported smoking status both prior to or during pregnancy.

| **Smoking Status** | **Controls (Normotensives)**  **n: Mean (median), [Range]** | ***p-value*** | **Cases (Preeclamptics)**  **n: Mean (median), [Range]** | ***p-value*** |
| --- | --- | --- | --- | --- |
| **Pre-Pregnancy** |  | | | |
| **Smoker** | n= 14: 4.0 (3.5), [1.7-8.7] |  | n= 14: 5.0 (3.5), [1.8-14.5] |  |
| **Non-smoker** | n=72: 3.4 (3.1), [0.52-7.7] | 0.45 | n= 72: 3.5 (3.0), [0.84-11.9] | 0.55 |
| **Pregnancy** |  | | | |
| **Smoker** | n= 7: 4.7 (4.2), [1.7-8.7] |  | n =4: 7 (5.4), [2.2-14.5] |  |
| **Non-smoker** | n= 79: 3.3 (3.1), [0.52-7.7] | 0.32 | n= 82: 3.5 (3.0), [0.84-11.9] | 0.44 |

+=Wilcoxon Rank Sum Test
