# Supplementary material for: Placental Cadmium Levels Are Associated with Increased Preeclampsia Risk
Source: PLoS One. 2015 Sep 30;10(9):e0139341. doi: 10.1371/journal.pone.0139341 (PMC4589375; doi:10.1371/journal.pone.0139341)
Supplement: S2 Table — (DOCX) [file pone.0139341.s003.docx]

S2 Table. Odds ratios and 95% Confidence Intervals (CI) for preeclampsia in relationship to placental Cd and interactive models for essential metals Se and Zn.

|  | **Model 1: Unadjusted Odds Ratio (95% CI)^a^** | **Model 2:**  **Odds Ratio**  **(95% CI)^b^** | **Model 3:**  **Odds Ratio**  **(95% CI)^c^** | **Model 4:**  **Odds Ratio**  **(95% CI)^d^** |
| --- | --- | --- | --- | --- |
| **Cd** | 1.1 (0.9-1.2) | 1.5 (1.1-2.2) | 1.2 (0.88-1.6) | 1.4 (1.01-2.2) |
| **Se** | 1.0 (0.99-1.0) | 1.0 (0.99-1.0) | 1.0 (1.0-1.0) | 1.0 (1.0-1.0) |
| **Zn** | 1.0 (1.0-1.0) | 1.0 (1.0-1.0) | 1.0 (1.0-1.0) | 1.0 (1.0-1.0) |
| **Cd x Se** |  |  |  |  |
| **Se ≤ median level^+^** | 1.1 (0.9-1.4) | 2.0 (1.1-3.5) | 1.5 (1.0-2.2) | 2.0 (1.1- 3.5) |
| **Se > median level^+^** | 0.98 (0.78-1.2) | 0.98 (0.5-1.9) | 0.79 (0.49-1.3) | 0.99 (0.84-1.9) |
| **Cd x Zn** |  |  |  |  |
| **Zn ≤ median level ^++^** | 0.97 (0.71-1.3) | 1.8 (0.8-3.9) | 1.2 (0.63-2.1) | 1.8 (0.84-3.9) |
| **Zn > median level ^++^** | 1.0 (0.84-1.2) | 1.3 (0.8-2.0) | 1.1 (0.79-1.6) | 1.3 (0.9-2.0) |

^a^ Model 1 represents the unadjusted model.

^b^ Model 2 correlated metals, maternal age, education, race, tobacco, alcohol use during pregnancy, public assistance recipient, gestational age, previous pregnancies, magnesium sulfate treatment, and periodontal disease treatment.

^c^ Model 3 correlated metals, maternal age, education, race, tobacco, alcohol use during pregnancy, public assistance recipient, previous pregnancies, magnesium sulfate treatment, and periodontal disease treatment (excluding gestational age).

^d^ Model 4 correlated metals, maternal age, education, race, tobacco, alcohol use during pregnancy, public assistance recipient, gestational age, previous pregnancies, magnesium sulfate treatment (excluding periodontal disease treatment).

+Se median=246 ng/g

++Zn median=8669 ng/g
